# Cross-sectional analysis of the Parkinson’s disease Non-motor International Longitudinal Study baseline non-motor characteristics, geographical distribution and impact on quality of life

**DOI:** 10.1038/s41598-021-88651-4

**Published:** 2021-05-05

**Authors:** Daniel J. van Wamelen, Anna Sauerbier, Valentina Leta, Carmen Rodriguez-Blazquez, Cristian Falup-Pecurariu, Mayela Rodriguez‐Violante, Alexandra Rizos, Y. Tsuboi, Vinod Metta, Roongroj Bhidayasiri, Kalyan Bhattacharya, Rupam Borgohain, L. K. Prashanth, Raymond Rosales, Simon Lewis, Victor Fung, Madhuri Behari, Vinay Goyal, Asha Kishore, Santiago Perez Lloret, Pablo Martinez-Martin, K. Ray Chaudhuri

**Affiliations:** 1 Department of Basic and Clinical Neuroscience, Institute of Psychiatry, Psychology and Neuroscience, King’s College London, London, UK; 2 Parkinson’s Foundation Centre of Excellence, King’s College Hospital, Denmark Hill, London, UK; 3 Department of Neurology, Donders Institute for Brain, Cognition and Behaviour, Radboud University Medical Centre, Nijmegen, The Netherlands; 4 Department of Neurology, Faculty of Medicine University Hospital Cologne, University of Cologne, Cologne, Germany; 5 National Center of Epidemiology, Center for Networked Biomedical Research in Neurodegenerative Diseases (CIBERNED), Carlos III Institute of Health, Madrid, Spain; 6 Department of Neurology, Faculty of Medicine, County Emergency Clinic Hospital, Transilvania University, Braşov, Romania; 7 Movement Disorders Unit, Instituto Nacional de Neurologia y Neurocirugía, Mexico, DF Mexico; 8 Department of Neurology, Faculty of Medicine, Fukuoka University, Fukuoka, Japan; 9 Chulalongkorn University Hospital, Bangkok, Thailand; 10 Formerly RG Kar Medical College and Institute of Neuroscience, Kolkata, India; 11 Nizam’s Institute of Medical Sciences, Hyderabad, India; 12 Center for Parkinson’s Disease and Movement Disorders Clinic, Vikram Hospitals, Bangalore, India; 13 Parkinson’s Disease and Movement Disorders Clinic, Bangalore, India; 14 University of Santo Tomas Hospital, Manila, Philippines; 15 ForeFront Parkinson’s Disease Research Clinic, Brain and Mind Centre, University of Sydney, Sydney, NSW Australia; 16 Movement Disorders Unit, Neurology Department, Westmead Hospital, Sydney, Australia; 17 Sydney Medical School, University of Sydney, Sydney, Australia; 18 Department of Neurology, Cardiothoracic and Neurosciences Centre, All India Institute of Medical Sciences, New Delhi, India; 19 Department of Neurology, All India Institute of Medical Sciences, New Delhi, India; 20 Sree Chitra Tirunal Institute for Medical Sciences and Technology, Trivandrum, India; 21 Biomedical Research Center, Interamerican Open University (CAECIHS-UAI), National Research Council (CONICET), Buenos Aires, Argentina; 22 Department of Physiology, School of Medicine, University of Buenos Aires, Buenos Aires, Argentina; 23 Center for Networked Biomedical Research in Neurodegenerative Diseases (CIBERNED), Carlos III Institute of Health, Madrid, Spain; 24 Department of Basic and Clinical Neuroscience, Institute of Psychiatry, Psychology and Neuroscience, De Crespigny Park, London, SE5 8AF UK

**Keywords:** Neuroscience, Diseases of the nervous system, Parkinson's disease, Neurological manifestations

## Abstract

Growing evidence suggests that non-motor symptoms (NMS) in Parkinson’s disease (PD) have differential progression patterns that have a different natural history from motor progression and may be geographically influenced. We conducted a cross-sectional analysis of 1607 PD patients of whom 1327 were from Europe, 208 from the Americas, and 72 from Asia. The primary objective was to assess baseline non-motor burden, defined by Non-Motor Symptoms Scale (NMSS) total scores. Other aims included identifying the factors predicting quality of life, differences in non-motor burden between drug-naïve and non-drug-naïve treated patients, and non-motor phenotypes across different geographical locations. Mean age was 65.9 ± 10.8 years, mean disease duration 6.3 ± 5.6 years, median Hoehn and Yahr stage was 2 (2–3), and 64.2% were male. In this cohort, mean NMSS scores were 46.7 ± 37.2. Differences in non-motor burden and patterns differed significantly between drug-naïve participants, those with a disease duration of less than five years, and those with a duration of five years or over (p ≤ 0.018). Significant differences were observed in geographical distribution (NMSS Europe: 46.4 ± 36.3; Americas: 55.3 ± 42.8; Asia: 26.6 ± 25.1; p < 0.001), with differences in sleep/fatigue, urinary, sexual, and miscellaneous domains (p ≤ 0.020). The best predictor of quality of life was the mood/apathy domain (β = 0.308, p < 0.001). This global study reveals that while non-motor symptoms are globally present with severe NMS burden impacting quality of life in PD, there appear to be differences depending on disease duration and geographical distribution.

## Introduction

Non-motor symptoms (NMS) are a key component of Parkinson’s disease (PD) and contribute significantly to reduced quality of life^1,2^. Although validated tools, such as the NMS Questionnaire (NMSQ)^3^ and Scale (NMSS)^4^, have been developed to address NMS burden in PD, not many studies have comprehensively looked at cross-sectional analysis of NMS data in large cohorts across different continents, the latter fact being relevant as intra-ethnic differences in reporting and patterns of NMS have been reported^5^. In recent years, there have been several cohort studies set up to map motor and non-motor progression of PD, such as the Parkinson Progression Marker Initiative (PPMI), the Discovery cohort and Tracking-PD, and COPPADIS to name a few^6,7^. These collaborative efforts are crucial to further understand the progression of PD and identify relevant biomarkers, which will help to address prognosis and management of this condition. The Non-Motor International Longitudinal Study (NILS) adds to this work, as it represents one of the few multicentre studies, which includes a detailed non-motor assessment.

The NILS (adopted to the UK national portfolio of research) was set up in 2011 and has a global reach with collaborative networks in Asia, USA, Latin America, Europe, as well as centres in Australia. The primary aim of NILS is to map non-motor characteristics in unselected PD populations, presenting to the clinician, using the NMSS (currently the most commonly used dedicated non-motor scale for PD worldwide)^8^, along with a range of motor and other non-motor validated scales and questionnaires. The extensive validation testing and associations of the NMSS with a broad range of NMS allows for an understanding of non-motor burden and progression in a real-life setting.

In this report, we aim to present the NILS initiative and currently available data of 1,607 participants with PD who have participated so far in NILS. Here, we describe baseline, one point in time, non-motor burden assessment in a cross-sectional design, and address predictors of quality of life. As secondary aims, we assessed the presence of non-motor differences in drug-naïve participants versus those with more advanced disease. Finally, we investigated whether non-motor profiles differ across PD populations as defined by their geographical location.

## Methods

Data were extracted from the prospective, longitudinal NILS, which was adopted by the National Institute of Health Research in the UK (UKCRN No: 10084). This initiative includes 34 centres across the world, currently containing data for over 1600 PD patients who had a baseline assessment. The study was authorised by the local ethics committees for each participating centre after ethical approval in Spain (Ethics committee for research and animal welfare from the Carlos III Institute of Health, CEI PI 02_2010) and the United Kingdom (NRES SouthEast London REC3, 10084, 10/H0808/141). All patients gave written consent prior to study procedures in accordance with the Declaration of Helsinki. The main inclusion criterion was a diagnosis of idiopathic PD according to the UK Brain Bank criteria. Exclusion criteria were (1) diagnosis of atypical Parkinsonism; (2) dementia (as per internationally accepted criteria)^9^; (3) inability for giving consent to participate in the study.

For the current analysis, we used data from patients whose data were entered between November 2011 and November 2019 (data extracted on the 1st of November 2019). We used data for the baseline assessment which included: sex, age at disease onset, disease duration, and Levodopa equivalent daily dose (LEDD). Patient-reported outcomes included Hospital Anxiety and Depression Scale (HADS)^10^, PD Questionnaire-8 item (PDQ-8) for quality of life^11^, Parkinson’s Disease Sleep Scale-version 1 (PDSS)^12^, and Visual Analogue Scale (VAS)^13^ for physical and mental fatigue scores. Clinician-based evaluations included Hoehn and Yahr (HY) staging^14^, Scales for Outcomes in PD (SCOPA) motor^15^, Mini Mental State Examination (MMSE)^16^, and NMSS scores^4^.

The primary aim of the current study was to evaluate the non-motor symptomatic burden, expressed by NMSS total scores. Other outcomes consisted of assessments of specific non-motor symptoms, measured through the abovementioned non-motor scales, and to determine which factors determined quality of life, as defined by the PDQ-8 instrument, in a multiple linear regression model. The latter was performed for the separate scale outcomes (regression model 1), as defined above, as well as for the separate domains of the NMSS (regression model 2).

Secondary aims of the study were: (1) to determine if NMS burden and profiles differed between drug naïve (DN) and treated patients, and (2) to determine whether NMS burden and profile differed according to geographical location. In order to assess the first secondary aim participants were divided into three groups: (1) de novo drug-naïve patients; (2) patients using dopaminergic medication with a disease duration of less than 5 years; and (3) patients using dopaminergic medication with a disease duration of five years or more. For the second secondary aims, we grouped patients based on the geographical location of their assessments. These groups depended on the location of the centre participating in NILS where the patient was assessed (continent and country of centres participating in NILS): (1) Europe (United Kingdom, Sweden, Germany, Italy, the Netherlands, Romania, Spain, Greece, and Israel); (2) Americas (Ecuador, Brazil, Venezuela, Argentina, United States, and Mexico); and (3) Asia (India and Japan).

### Statistical analyses

As the scores of the NMSS scale data were not normally distributed (as determined through Kolmogorov–Smirnov test), we used the Kruskal–Wallis test to evaluate the differences between groups. Corrections for statistically significant demographic group differences were performed using a Quade’s rank analysis. In order to select which variables to include in a multiple linear regression model to assess which factors contributed to PDQ-8 scores, we first performed univariate analyses between the different baseline scale assessments, as mentioned above, and PDQ-8 scores. Any factor that was significantly associated (p < 0.05) with PDQ-8 scores was selected as a variable for the multiple linear regression model with PDQ-8 scores as dependent variable, and the identified factors in the univariate model as independent variables. To determine which NMSS domain contributed most to quality of life, we performed a second multiple linear regression model with PDQ-8 as the dependent variable, and the separate NMSS domains identified as having a significant association with PDQ-8 scores in a univariate model as independent variables.

The significance threshold was set at ≤ 0.05 and a Benjamini–Hochberg procedure was used in in case of multiple comparisons. All data were analysed using SPSS Version 25 (IBM SPSS Statistics for Windows (Version 25.0. Armonk, NY: IBM Corp.). Data are represented as mean ± standard deviation, number (percentage), median (25th-75th percentile), or standardised β values, unless otherwise specified.

## Results

### Patient cohort

Table 1 provides an overview of the demographics for the 1,607 patients with their baseline assessment. Within the entire cohort, 64.2% were male and 35.8% female, age was 65.9 ± 10.80 years, disease duration 6.3 ± 5.6 years, and median Hoehn and Yahr stage 2 (2–3). Mean LEDD was 551.4 ± 582.7 mg and 191 (11.8%) of participants were drug-naïve. SCOPA-motor scores were 11.4 ± 6.0 for motor scores, 5.3 ± 3.5 for activities of daily living, and 2.1 ± 2.7 for motor complications. In the entire cohort, all patients had at least one NMS. The NMSS total score was 46.7 ± 37.2 in the entire cohort (severe non-motor burden; Table 1). For only 5 patients was the NMSS score missing, showing a good level of data capture in our study. The scores for other non-motor scales are provided in Table 1.

**Table 1:** Demographic data for the entire cohort and across different geographical sites of assessment.

Parameters	Entire cohort (n = 1607)	Europe (n = 1327)	Americas (n = 208)	India and Japan (n = 72)	P^1^	P^2^
Age	65.89 ± 10.80	66.31 ± 10.77	62.94 ± 10.31	66.56 ± 11.43	< 0.001	N/A
Sex (M/F)	1030/574 (64.2/35.8%)	859/465 (64.9/35.1%)	123/85 (59.1/40.9%)	48/24 (66.7/33.3%)	0.249	0.299
Disease duration	6.34 ± 5.59	6.06 ± 5.41	8.28 ± 6.49	5.89 ± 4.97	< 0.001	N/A
Hoehn and Yahr	2 (2–3)	2 (2–3)	2 (2–3)	3 (2–3.75)	< 0.001	0.220
LEDD	551.38 ± 582.71	559.21 ± 590.26	589.27 ± 589.79	297.61 ± 298.45	< 0.001	0.228
Drug-naïve Oral medication Apomorphine Intrajejunal levodopa Deep brain stimulation	191 (11.8%) 277 (82.8%) 25 (1.6%) 10 (0.6%) 51 (3.2%)	171 (12.9%) 1070 (80.6%) 25 (1.9%) 10 (0.8%) 51 (3.8%	19 (9.1%) 189 (90.9%) 0 (0.0%) 0 (0.0%) 0 (0.0%)	2 (2.8%) 70 (97.2%) 0 (0.0%) 0 (0.0%) 0 (0.0%)	N/A	N/A
**SCOPA-motor**						
Motor Activities of daily living Complications	11.40 ± 6.02 5.26 ± 3.52 2.08 ± 2.73	11.18 ± 5.89 5.19 ± 3.55 1.99 ± 2.73	11.41 ± 6.41 5.20 ± 3.49 2.45 ± 2.73	15.26 ± 6.08 6.56 ± 2.89 2.67 ± 2.61	< 0.001 < 0.001 0.001	0.125 0.192 0.940
**NMSS**^**a,b**^ 1. Cardiovascular 2. Sleep/fatigue^a,b^ 3. Mood/apathy 4. Perceptual 5. Memory 6. Gastrointestinal 7. Urinary^a,b,c^ 8. Sexual^b,c^ 9. Miscellaneous^b,c^	46.70 ± 37.17 1.69 ± 3.01 8.72 ± 7.33 8.37 ± 12.19 1.29 ± 3.31 4.83 ± 6.70 4.57 ± 5.82 7.40 ± 8.68 2.97 ± 5.67 6.87 ± 7.23	46.44 ± 36.33 1.74 ± 3.00 8.93 ± 7.43 8.00 ± 11.74 1.26 ± 3.24 4.80 ± 6.66 4.56 ± 6.66 7.51 ± 8.62 2.75 ± 5.44 6.89 ± 7.18	55.27 ± 42.80 1.60 ± 3.29 8.43 ± 7.21 11.21 ± 15.34 1.56 ± 3.98 5.68 ± 7.57 5.00 ± 6.72 8.44 ± 9.54 4.99 ± 7.20 8.36 ± 7.85	26.60 ± 25.14 1.06 ± 2.03 5.56 ± 4.70 6.81 ± 8.60 1.07 ± 2.26 2.81 ± 3.88 3.54 ± 4.26 2.35 ± 4.40 1.26 ± 2.84 2.14 ± 2.99	< 0.001 0.236 0.001 0.058 0.397 0.103 0.939 < 0.001 < 0.001 < 0.001	** < 0.001** 0.068 **0.020** 0.299 0.440 0.220 0.754 ** < 0.001** **0.020** ** < 0.001**
**HADS**^**a,b**^ Anxiety^a,b,c^ Depression^b,c^	11.88 ± 7.45 6.29 ± 4.30 5.59 ± 3.96	11.24 ± 7.29 5.95 ± 4.21 5.29 ± 3.86	13.62 ± 7.55 7.49 ± 4.26 6.13 ± 3.98	18.32 ± 6.05 8.89 ± 4.52 9.43 ± 3.57	< 0.001 < 0.001 < 0.001	** < 0.001** ** < 0.001** ** < 0.001**
PDSS^a,b^	103.95 ± 29.14	106.90 ± 26.77	96.45 ± 28.82	74.06 ± 45.39	< 0.001	** < 0.001**
MMSE^a^	27.96 ± 2.84	28.06 ± 2.90	27.40 ± 2.49	27.96 ± 2.65	< 0.001	**0.008**
**VAS fatigue**						
Mental Physical	34.33 ± 27.17 39.67 ± 26.07	34.65 ± 26.75 40.67 ± 25.52	30.81 ± 30.93 32.80 ± 30.02	38.75 ± 21.81 41.26 ± 20.50	0.003 < 0.001	0.830 0.125
PDQ-8^b,c^	21.96 ± 15.78	20.85 ± 15.66	23.73 ± 14.55	36.81 ± 13.55	< 0.001	** < 0.001**

Data presented as mean ± standard deviation, except for Hoehn and Yahr stage (median (25th-75th percentile)), or number (percentage). 1—uncorrected p-value; 2—p-value after Quade’s rank analysis correcting for age and disease duration and after correction for multiple testing using a Benjamini Hochberg procedure. Post-hoc analyses (corrected values using Benjamini–Hochberg procedure): ^a^difference between Europe and Americas (p < 0.05); ^b^difference between Europe and India/Japan (p < 0.05); ^c^difference between Americas and India/Japan (p < 0.05).

P-values in bold are significant (p ≤ 0.05).

### Non-motor burden in drug naive patients

In the NILS cohort, 191 participants were de novo patients. In addition, 650 participants had a disease duration of less than five years, and 739 of 5 years or over (for 27 patients disease duration was not available). Demographic data and distribution of non-motor scores for the NMSS and other scales used for these three groups are provided in Table 2.

**Table 2:** Non-motor burden and profile differences between drug-naïve and medicated participants with Parkinson’s disease.

Parameters	1. Drug-naïve patients (n = 191)	2. Disease duration < 5 years (n = 650)	3. Disease duration ≥ 5 years (n = 739)	P^1^	P^2^	Post-hoc groups 1–2	Post-hoc groups 2–3
Age	66.09 ± 11.68	65.17 ± 11.19	66.30 ± 9.81	0.197	0.205	N/A	N/A
Sex (M/F)	116/75 (60.7%/39.3%)	417/233 (64.2%/35.8%)	479/258 (64.1%/35.9%)	0.550	0.550	N/A	N/A
Disease duration	1.27 ± 1.18	2.69 ± 1.41	10.87 ± 5.09	N/A	N/A	N/A	N/A
Hoehn and Yahr	2.0 (1.0–2.0)	2.0 (1.0–2.0)	3.0 (2.0–3.0)	< 0.001	** < 0.001**	** < 0.001**	** < 0.001**
LEDD	0.00 ± 0.00	420.54 ± 361.38	807.89 ± 664.53	< 0.001	** < 0.001**	** < 0.001**	** < 0.001**
**SCOPA-motor** Motor Activities of daily living Complications	13.65 ± 7.52 10.00 ± 5.11 3.65 ± 3.01 0.00 ± 0.00	15.68 ± 8.34 10.28 ± 5.26 4.32 ± 2.98 1.08 ± 1.79	22.61 ± 10.97 12.67 ± 6.51 6.45 ± 3.60 3.49 ± 2.99	< 0.001 < 0.001 < 0.001 < 0.001	** < 0.001** ** < 0.001** ** < 0.001** ** < 0.001**	**0.020** 0.499 **0.026** ** < 0.001**	** < 0.001** ** < 0.001** ** < 0.001** ** < 0.001**
**NMSS** 1. Cardiovascular 2. Sleep/fatigue 3. Mood/apathy 4. Perceptual 5. Memory 6. Gastrointestinal 7. Urinary 8. Sexual 9. Miscellaneous	39.36 ± 32.90 1.47 ± 2.64 7.24 ± 6.72 9.07 ± 13.41 0.53 ± 1.68 4.05 ± 5.60 3.16 ± 4.94 5.59 ± 7.22 2.61 ± 5.31 5.63 ± 6.26	39.67 ± 33.37 1.44 ± 2.75 7.64 ± 6.60 7.51 ± 11.51 1.00 ± 2.63 4.33 ± 6.52 3.50 ± 4.68 6.29 ± 7.83 2.23 ± 4.93 5.74 ± 6.59	54.70 ± 39.68 1.97 ± 3.30 10.00 ± 7.86 8.94 ± 12.35 1.74 ± 4.02 5.49 ± 7.08 5.81 ± 6.58 8.85 ± 9.45 3.74 ± 6.25 8.14 ± 7.79	< 0.001 0.020 < 0.001 0.005 < 0.001 < 0.001 < 0.001 < 0.001 < 0.001 < 0.001	** < 0.001** **0.022** ** < 0.001** **0.006** ** < 0.001** ** < 0.001** ** < 0.001** ** < 0.001** ** < 0.001** ** < 0.001**	0.962 0.446 0.798 0.086 0.657 0.979 0.345 0.343 0.648 0.994	** < 0.001** **0.016** ** < 0.001** **0.003** **0.001** ** < 0.001** ** < 0.001** ** < 0.001** ** < 0.001** ** < 0.001**
**HADS** Anxiety Depression	10.61 ± 7.57 5.74 ± 4.42 4.86 ± 3.91	11.52 ± 7.28 6.21 ± 4.34 5.31 ± 3.80	12.55 ± 7.50 6.52 ± 4.21 6.02 ± 4.07	0.001 0.016 < 0.001	**0.001** **0.018** ** < 0.001**	0.295 0.343 0.265	0.304 **0.005** **0.031**
PDSS	109.08 ± 27.12	106.92 ± 29.88	100.16 ± 28.61	< 0.001	** < 0.001**	0.604	** < 0.001**
MMSE	28.27 ± 2.64	28.20 ± 2.46	27.66 ± 3.18	0.002	**0.003**	**0.020**	**0.006**
**VAS fatigue**							
Mental Physical	31.32 ± 29.19 36.79 ± 28.66	32.80 ± 26.31 38.15 ± 25.62	36.45 ± 27.06 42.00 ± 25.55	0.005 0.005	**0.006** **0.006**	0.430 0.693	**0.023** **0.043**
PDQ-8	6.59 ± 5.97	7.72 ± 5.86	10.33 ± 6.40	< 0.001	** < 0.001**	**0.025**	** < 0.001**

Data presented as mean ± standard deviation, except for Hoehn and Yahr stage (median (25th–75th percentile)), or number (percentage). 1—uncorrected p-value; 2—p-value after correction for multiple testing using a Benjamini Hochberg procedure.

P-values in bold are significant (p ≤ 0.05).

**Figure 1:**
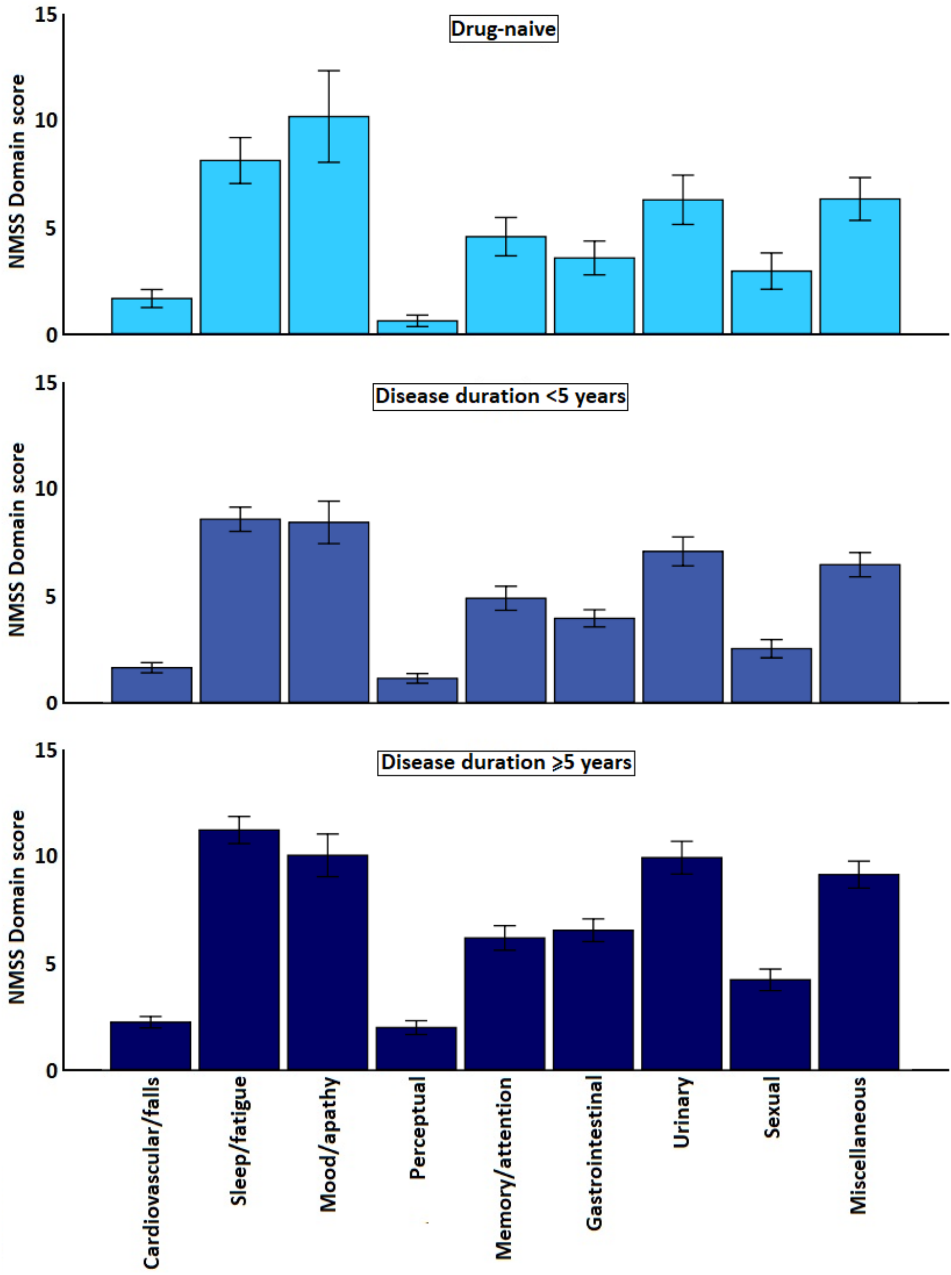
Distribution of specific non-motor domains of the Non Motor Symptoms Scale (NMSS) across drug-naïve and non-drug-naïve participants with Parkinson’s disease. Data presented as mean; bars represent the 95% confidence intervals. All NMSS domain scores were significantly higher in participants with a disease duration of ≥ 5 years compared to those with a duration < 5 years (p ≤ 0.016), but no domains between drug-naïve participants and those with a disease duration of < 5 years (p ≥ 0.086).

When comparing these three groups, after correction for multiple testing, we noticed that despite the difference in disease duration, no differences were observed in age (p = 0.205) or distribution of sex (p = 0.550). There were statistically significant differences in all non-motor scale scores across the three groups (p ≤ 0.018; Table 2) with NMSS scores being 39.4 ± 32.9 in the DN patients, 39.7 ± 33.4 in those non-DN patients with a disease duration of less than five years, and 54.7 ± 39.7 in those non-DN with a duration of five years and over (Fig. 1). However, a post-hoc analysis, revealed that the most pronounced difference in the NMSS and other non-motor scale scores (except for the HADS total scores) was observed between participants with a disease duration of less than 5 years and those with a duration of five years or over (p ≤ 0.043; Table 2). When comparing DN patients to those with a disease duration of less than 5 years and on dopaminergic medication, the only significant differences could be observed for MMSE scores (p = 0.020) and PDQ-8 summary index scores (p = 0.025; Table 2).

### Geographical distribution

**Figure 2:**
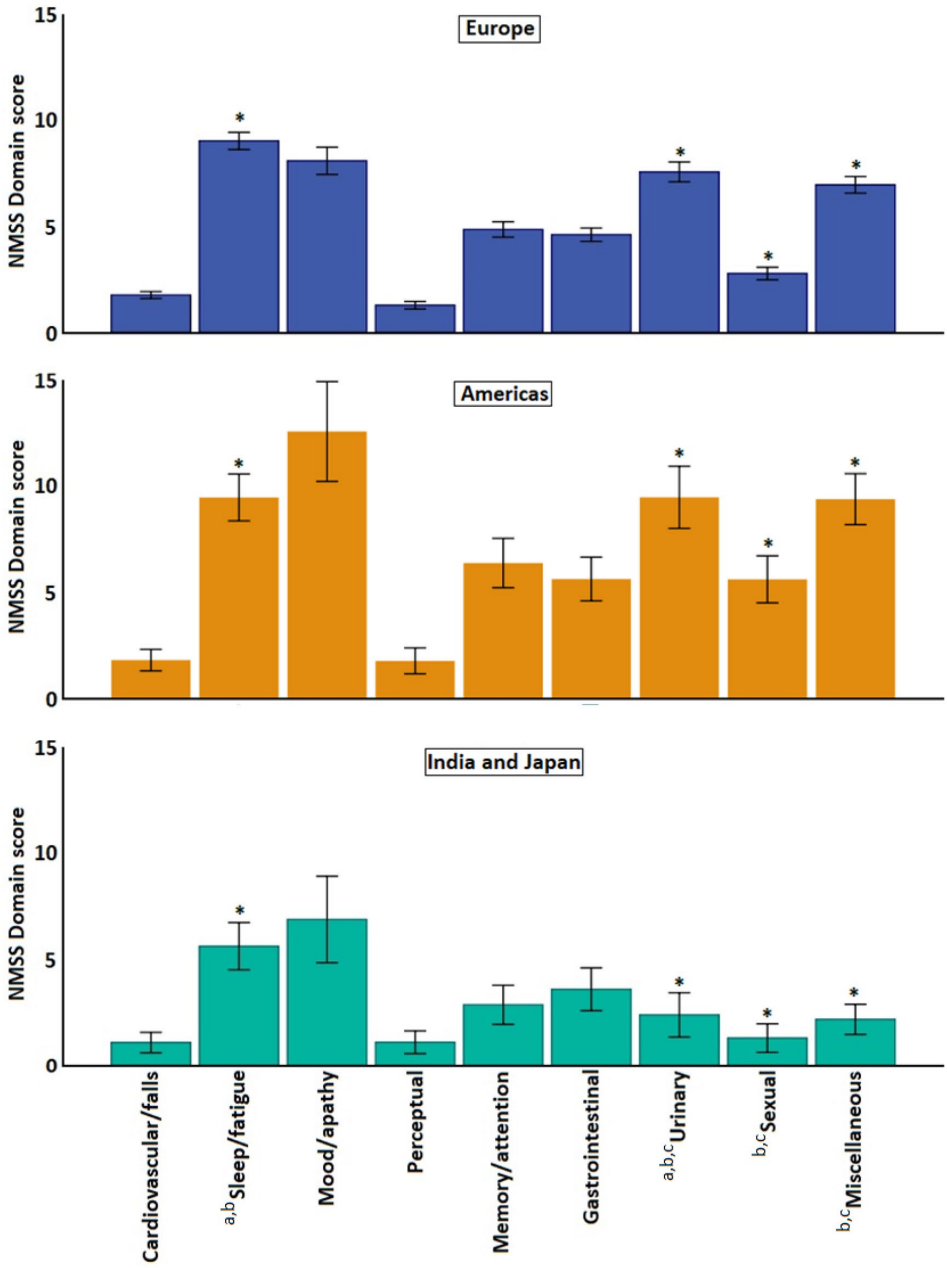
Distribution of specific non-motor domains of the Non Motor Symptoms Scale across different geographical regions in participants with Parkinson’s disease. Data presented as mean; bars represent the 95% confidence intervals; * significant (p < 0.05) group differences. Post-hoc analyses (corrected values using Benjamini–Hochberg procedure): **(a)** difference between Europe and Americas (p < 0.05); **(b)** difference between Europe and India/Japan (p < 0.05); **(c)** difference between Americas and India/Japan (p < 0.05).

In addition to the differences between DN and non-DN patients we also observed significant differences between participants depending on geographical location. The NMSS total scores were highest in patients from the Americas (55.3 ± 42.8), and lowest in Indian and Japanese patients (26.6 ± 25.1) (p < 0.001; Table 1). Given that significant differences in age and disease duration were observed between the different cohorts, we performed a correction for these factors using a Quade’s analysis. After correction, the other demographic variables and motor scores no longer differed between the groups (p ≥ 0.125). However, the differences in the total NMSS scores (p < 0.001) and in some specific domain scores persisted, with significant differences in the sleep/fatigue, urinary, sexual, and miscellaneous domains (p ≤ 0.020; Table 1; Fig. 2). In the entire cohort, the highest domain scores were observed for domain 2 (sleep/fatigue), domain 7 (urinary), and domain 9 (miscellaneous). These domains also had the highest scores in the European patients. In the cohorts form the Americas, India and Japan, on the other hand, additional high domain burden was also observed for domain 3 (mood/apathy) (Table 1; Fig. 2).

We also observed significant differences in other non-motor scales across the patients from different continents, persisting after the Quade’s rank correction for age and disease duration. These included differences in the PDSS and HADS scores, with worst scores in the Indian and Japanese patients and the best scores in the European cohort (Table 1; Fig. 2). Also for MMSE significant geographical differences were observed (Table 1); however the absolute score difference between groups was small.

Given the relatively small sample size of the India/Japan cohort, as a sensitivity analysis, we decided to repeat a separate analysis using Mann–Whitney U test, comparing only the cohorts from Europe and Americas. Here, the differences observed in the main analyses for non-motor outcomes (NMSS total and domain 2, 7, 8, and 9 scores; HADS total and anxiety and depression scores; PDSS; and PDQ-8; p < 0.001) remained significant, except for MMSE (p = 0.82) (data not shown).

### Quality of life

Before performing the regression models described below, we performed outlier detection to serve as a simple goodness of fit diagnostics. This showed 0.1% outliers for age, 0.3% for disease duration, 0.6% for LEDD, 0.4% for NMSS, and no outliers for SCOPA motor scores. For the NMSS domains outliers ranged between 0.0% and 0.7% for the separate domains, except for the perceptual/hallucinations domain where it was 1.1%. Given the low number of outliers all below analyses were performed without omission of outliers. In addition, in order to determine possible collinearity, before variables were entered in the linear regression model, we performed interdomain correlations between the NMSS domains. This showed that rho values were in the 0.1 to 0.3 range with only one value being higher at 0.45, and, as such, we feel that collinearity was not a problem in the regression model.

For the entire cohort of 1,607 patients the multiple regression model (R^2^ = 0.445, p < 0.001) showed that the best predictors of quality of life were SCOPA motor (β = 0.413, p < 0.001) and total NMSS scores (β = 0.360; p < 0.001; Table 3). Also here, differences could be observed depending on the geographical location. Multiple linear regression models were repeated for each geographical location on the significant variables that were associated with the PDQ-8 in the univariate models (Tables 3 and 4). In Europe, the two most important predictors of QoL, in almost equal proportion, were the SCOPA motor scores (β  = 0.421, p < 0.001) and the NMSS scores (β  = 0.386, p < 0.001), but in the cohort from the Americas NMSS scores contributed more than SCOPA motor scores (Table 3). In the Asian patients no significant predictors of QoL in the multiple linear regression could be observed, despite a 0.77 post-hoc power.

**Table 3:** Regression model with predictors of quality of life, as measured by the PDQ-8 scale.

	Europe (n = 1327)		Americas (n = 208)		India and Japan (n = 72)		Entire cohort (n = 1607)	
Model fit	R^2^ = 0.496, p < 0.001		R^2^ = 0.407, p < 0.001		R^2^ = 0.147, p = 0.012		R^2^ = 0.445, p < 0.001	
Parameters	β*	p	β*	p	β*	p	β*	p
Age	N/A	N/A	N/A	N/A	−0.177	0.167	N/A	N/A
Disease duration	0.003	0.902	0.154	**0.013**	N/A	N/A	0.027	0.224
LEDD	−0.006	0.780	−0.039	0.507	0.195	0.136	−0.049	**0.021**
SCOPA	0.421	** < 0.001**	0.240	**0.001**	N/A	N/A	0.413	** < 0.001**
NMSS	0.386	** < 0.001**	0.411	** < 0.001**	0.022	0.052	0.360	** < 0.001**

*LEDD* levodopa equivalent daily dose, *SCOPA* Scales for Outcomes in Parkinson’s disease, *NMSS* Non Motor Symptoms Scale, *N/A* not applicable (factor not associated with PDQ-8 scores in a univariate model), β* standardised β. Only variables which were significantly associated with PDQ-8 scores in a univariate model were included in the respective analyses.

P-values in bold are significant (p ≤ 0.05).

**Table 4:** Regression model with Non Motor Symptom Scale domains as predictors of quality of life (measured by the PDQ-8 scale).

Parameters	Europe (n = 1327)		Americas (n = 208)		India and Japan (n = 72)		Entire cohort (n = 1607)	
Model	R^2^ = 0.651, p < 0.001		R^2^ = 0.390, p < 0.001		R^2^ = 0.100, p = 0.064		R^2^ = 0.357, p < 0.001	
NMSS domains	β*	p	β*	p	β*	p	β*	p
Cardiovascular/falls	0.076	** < 0.001**	0.016	0.805	N/A	N/A	0.070	** < 0.001**
Sleep/fatigue	0.173	**0.001**	0.065	0.354	0.047	0.758	0.143	**0.002**
Mood/apathy	0.288	** < 0.001**	0.424	** < 0.001**	0.267	0.080	0.308	** < 0.001**
Perceptual/hallucinations	0.084	** < 0.001**	0.041	0.511	N/A	N/A	0.077	** < 0.001**
Memory/attention	0.154	** < 0.001**	0.042	0.558	N/A	N/A	0.125	**0.001**
Gastrointestinal	0.110	** < 0.001**	0.075	0.238	N/A	N/A	0.109	** < 0.001**
Urinary	0.037	0.127	− 0.058	0.372	N/A	N/A	0.100	** < 0.001**
Sexual	0.004	0.860	0.080	0.200	N/A	N/A	0.002	0.913
Miscellaneous	0.039	0.087	0.184	**0.003**	0.046	0.713	0.029	0.186

*NMSS* Non Motor Symptoms Scale, *N/A* not applicable (factor not associated with PDQ-8 scores in a univariate model); β* standardised β. Only domains which were significantly associated with PDQ-8 scores in a univariate model were included in the respective analyses.

P-values in bold are significant (p ≤ 0.05).

For the individual NMSS domains, we found that domain 3 (mood/apathy) (β  = 0.308, p < 0.001) and domain 2 (sleep/fatigue; β  = 0.143, p = 0.002) were the best predictors for QoL (Table 4). In the Indian and Japanese populations, no significant NMSS domain predictor for QoL could be identified, whereas in the cohort from the Americas the mood/cognition and miscellaneous domains were predictive of QoL (β  = 0.424, p < 0.001, and β  = 0.184, p = 0.003, respectively). The latter was also true for European patients, with additional predictors in the form of the NMSS cardiovascular/falls, sleep/fatigue, perceptual/hallucinations, attention/memory and gastrointestinal domains (Table 4).

## Discussion

To our knowledge, with over 1600 patients, the current study represents one of the largest cross-sectional studies assessing non-motor symptoms in multi-national PD patients. The key findings were:

(a) A universal presence of NMS irrespective of age, motor severity, disease duration and geographical site of assessment, with an overall severe non-motor burden;

(b) Most frequently, the highest domain burden was observed for the sleep/fatigue, mood/apathy, urinary, and miscellaneous domains;

(c) NMS impacted significantly on quality of life, which was best predicted by the mood/apathy and sleep/fatigue domains of the NMSS;

(d) The burden of NMS increased with disease duration, starting after five years since diagnosis;

(e) Geographical non-motor burden differences appear to exist, with the highest NMSS scores in the Americas and the lowest in Asia, although with marked differences in the burden of specific non-motor domains, including sleep/fatigue, urinary, and miscellaneous domains.

### Quality of life

A large body of evidence has confirmed the marked importance of NMS for QoL in PD patients. In our cohort, quality of life was best predicted by motor scores (measured through the SCOPA motor scale) and NMSS total scores. Also, at least at the level of the entire cohort, LEDD predicted quality of life, but had a very low β value. In the latter, especially the mood/apathy and sleep/fatigue domains showed a clear negative impact on QoL. However, we were able to show that, similar to the effect on non-motor burden, geographical location appears to have an effect on which of the specific NMS are most likely to impact QoL. Whereas mood and sleep appear to have the largest impact in European patients, in the Indian and Japanese patients, no single NMS was a significant predictor of QoL in our multiple linear models, perhaps due to the small sample size. These findings contrast to those previously observed in Asian populations. For example, a cross-sectional study from China demonstrated that sleep/fatigue, mood, gastrointestinal, urinary and, miscellaneous had a negative impact on QoL^17^. Along these lines, Prakash and colleagues showed that sleep, mood, and attention disorders have a significant negative impact on QoL in patients from Singapore^18^. Our findings in the European cohort largely mimic previous studies, where the “PRIAMO” multicentre study conducted in Italy reported that especially apathy/fatigue, attention/memory, and psychiatric symptoms had a negative impact on QoL^19^.

### NMS burden and profiles between drug naïve (DN) and treated patients

This large-scale study, even in the light of the current cross-sectional design, confirmed that non-motor burden increases with disease duration. However, it appears that this progressive worsening is neither linear, as demonstrated by the equal non-motor burden in drug-naïve patients and medicated patients with a disease duration of less than five years, nor evenly distributed across different NMS. The latter is exemplified by the relatively stable burden of e.g. cardiovascular and depressive symptoms across the different groups in our analyses. Our current findings, and the observation that NMS burden is dependent on disease duration, are illustrated by several other large-scale non-motor studies. For example, in the original NMSS validation study, Chaudhuri and colleagues reported a higher mean NMSS score of 56 (at a mean disease duration 6.4 years), while a study evaluating the NMSS scale in Chinese patients revealed a lower score of 31 (at a mean disease duration of 5.4 years). Similarly, another Chinese cohort of 117 PD patients with a mean disease duration of 3.9 years, showed that NMSS total scores were 35.1 at baseline^20^. On the other hand, Antonini and colleagues found that in 707 patients the NMS burden was 21.2 (moderate NMS burden^21^) in patients with a mean HY stage of 1.97^22^. Finally, Prakash et al. showed a moderate NMS burden in their cohort of PD patients with a disease duration of 5.8 years (NMSS total score 27.5)^18^. As such, these studies demonstrate that disease duration is an important factor for non-motor burden.

### NMS burden and profiles according to geographical distribution

In our cohort, we observed marked differences in NMS burden between different geographical locations, and also in the NMS patterns in terms of which symptoms have the highest burden across different locations. Even though these findings did not take into account the ethnic background of the populations studied, but merely the country of assessment, they still provide a clue into the effect on non-motor burden of ethnicity and other factors that differ between geographical locations. These NMS differences have been highlighted before and our current findings are largely in line with previously published studies. A recent literature review on NMS assessed with the NMSS in Asian cohorts concluded that symptoms within the domains sleep/fatigue, attention/memory, and mood/apathy appear to be the most prevalent^23^. Kedhr and colleagues showed that the most commonly reported NMS in Egyptian PD patients were gastrointestinal problems, sleep/fatigue, mood/apathy, and cognition^24–26^. A similar pattern was observed in Moroccan PD patients^27^. Interestingly, four Asian studies (including patients from India, Korea and China) reported the miscellaneous domain among the three most prevalent NMSS domains^23^. In line with our findings in Indian and Japanese PD patients, Cheon and colleagues showed, in 74 Korean PD patients that the most common NMS were nocturia, restless legs, constipation, depressive feelings, orthostatic hypotension, and memory problems^28^.

Unlike our cohort, many studies performed in Asia have shown a very high prevalence of sexual problems (up to 84.7%)^29,30^. Other studies have shown that in India and Singapore sleep problems and fatigue are very common (83.9–89.7%), coinciding with the high NMSS domain burden for these symptoms in our cohort^31,32^. Even though we did not directly study the effect of ethnicity, our findings provide a basis for further research into this topic. Future studies should also aim to address differences in NMS profiles and burden across different ethnic Asian populations. For this the newly developed Movement Disorder Society Non-Motor Scale could prove beneficial as it contains several NMS that are not in included in the NMSS^33^.

### Limitations

As with other studies, limitations in our study need to be acknowledged. We did not have a control group of healthy subjects to compare NMS burden against, as it known that also otherwise healthy people experience NMS. Also, the findings in our cohort may have been partly confounded by medication use as we included patients through all stages of the disease, although most were in HY stage 2 or 3. Also, a longitudinal design in order to study the development of non-motor burden across different disease stages would have been preferable instead of the current cross-sectional design. In addition, one of our main findings was the difference in non-motor burden across different geographical sites, but we lacked information on the ethnic background of patients, and the country of assessment did not define a person’s ethnic background. This should be addressed in future studies where clearly defined information regarding ethnicity background should be available. Also, the sample of Indian and Japanese patients was considerably smaller than our European cohort. However, we still consider our findings clinically useful and reflect clinical reality. Moreover, we feel that the limitations to this study are counterbalanced by the large sample size, the structured non-motor assessments, and the inclusion of patients at all stages of the disease with a broad range in disease duration.

In summary, we observed that NMS are very common across all stages of PD, and that average burden of NMS is moderate, but appears to increase to severe in more advanced disease. In our cohort, the most common NMS in PD were sleep/fatigue, urinary, and miscellaneous problems, along with high HADS and low PDSS scores (the latter indicating poor quality of sleep). However, there appear to be geographical differences, with higher NMSS scores in the Americas, and lower scores in India and Japan, along with differences in domain and specific NMS burden. These exploratory data can serve as a basis for future research into the effect of geographical location and ethnicity of NMS burden. The NMS with the strongest impact on quality of life were depression, anxiety and sleep. The data from this large cohort of PD should be followed up by longitudinal data looking at the specific progression of NMS in PD and the effect of ethnicity on NMS.

## Data Availability

The data describes in this study will be made available by the corresponding author upon reasonable request.
